# Social network data of Swiss farmers related to agricultural climate change mitigation

**DOI:** 10.1016/j.dib.2021.106898

**Published:** 2021-02-19

**Authors:** Cordelia Sophie Kreft, Mario Angst, Robert Huber, Robert Finger

**Affiliations:** a Agricultural Economics and Policy Group, ETH Zurich, Zurich Switzerland; b Swiss Federal Institute for Forest, Snow and Landscape Research WSL

**Keywords:** Farmers’ social networks, Agricultural climate change mitigation, Grassroots initiative, Social learning, Network canvas software, Switzerland

## Abstract

We present social network data of Swiss farmers, focusing on exchange and advice relations regarding agricultural climate change mitigation. The data were generated via face-to-face interviews in 2019 using the survey software Network Canvas (https://networkcanvas.com). We interviewed 50 farmers, with 25 of these participating in a regional climate protection initiative in Switzerland as well as 25 farmers located in the same region who did not participate in the initiative. Farmers were asked to indicate the persons with whom they regularly exchanged on topics related to climate change and mitigation in agriculture. The farmers assessed the type and strength of their relationships and were asked to rate the knowledge of their contacts regarding climate change mitigation. We also collected data on the perceived influence of farmers and other persons on farming decisions. Information on farmers’ adoption of climate change mitigation measures and behavioural characteristics was collected in a previous online survey. Farm characteristics were obtained from census data.

## Specifications Table

**Table:** 

Subject	Agricultural Economics; Climate Change
Specific subject area	Farmers’ social networks with regard to agricultural climate change mitigation
Type of data	Table
How data were acquired	Face-to-face interviews using Network Canvas Software on tablets, online surveys, farm census data
Data format	Raw and partly filtered (for reasons of confidentiality)
Parameters for data collection	Interviews were conducted with farmers participating in a regional climate protection initiative as well as with non-participating farmers in the same region
Description of data collection	Interviews with farmers were scheduled by telephone and conducted face-to-face on site (usually on the farm) by four trained interviewers. Questions were asked using the network survey tool Network Canvas installed on tablets. Farmers were asked to directly place named contacts on different sociograms on the tablet. The data was anonymized.
Data source location	Region of Zürcher Weinland, Canton Zurich, Switzerland
Data accessibility	Data is accessible via ETH Zürich Research Collection: http://hdl.handle.net/20.500.11850/458053

## Value of the Data

• The data provides detailed information on farmers’ social networks and their potential role in reduction of agricultural greenhouse gas emissions. The combination with farm census data as well as data from a previous online survey on adoption of mitigation measures and behavioural characteristics represents a comprehensive data basis.
• The data allows for in-depth insights in famers’ decision-making in the context of climate change mitigation.
• The data can be used to analyze the role of social networks in adoption of climate change mitigation measures. A wide range of behavioural factors and farm characteristics allows for a comprehensive set of control variables.
• The data enables the use of social network analysis techniques in combination with econometric analyses and/or mathematical modeling.

## 1 Data Description

We collected data on social networks of farmers with regard to climate change mitigation in the region of Zürcher Weinland, Canton Zurich, Switzerland. Interviews were conducted face-to-face with 25 farmers participating in the bottom-up climate protection initiative AgroCO2ncept Flaachtal [1] (hereafter: AgroCO2ncept) as well as with 25 farmers in the same region who were not participating in the initiative. The two interview questionnaires were slightly different for AgroCO2ncept participants and non-participants, e.g. participants were specifically asked about the project. Both questionnaires, all resulting datasets as well as the codebooks describing the variables are available through the ETH Zürich Research Collection: http://hdl.handle.net/20.500.11850/458053.

10 datasets resulted from the interviews as listed in Table 1.

**Table 1:** Overview of datasets.

Farmers interviewed	Dataset ID	Data content	Complete datasets available	Data file name
AgroCO2ncept participants (*n* = 25)	1	Farmers’ personal attributes derived from interviews (“ego attributes”)	25	Atts_agroconcept_int.csv
	2	Additional farmers’ attributes from online survey and census data	24 (ID 16 has not answered survey)	Atts_agroconcept_survey.csv
	3	Ties between AgroCO2ncept farmers only (complete network)	25 senders, 25 receivers	Edges_agroconcept_complete.csv
	4	Ties between AgroCO2ncept farmers as well as with non-members (external contacts)	25 senders, 53 receivers	Edges_agroconcept_and_external_contacts.csv
	5	Influential people in the region named by AgroCO2ncept farmers	24 senders, 32 receivers	Influence_agroconcept.csv
Farmers not participating in AgroCO2ncept (*n* = 25)	6	Farmers’ personal attributes derived from interviews (“ego attributes”)	25	Atts_nonpart_int.csv
	7	Additional farmers’ attributes from online survey and census data	22	Atts_nonpart_survey.csv
	8	All ties named by non-participants	25 senders, 74 receivers	Edges_nonpart_all.csv
	9	Ties from non-participants to AgroCO2ncept members only	25 senders, 30 receivers (including some co-managers of AgroCO2ncept farms)	Edges_nonpart_to_agroconcept.csv
	10	Influential people in the region named by non-participants	16 senders, 25 receivers	Influence_nonagroconcept.csv

The presented data contain information on farmers’ social ties to AgroCO2ncept members as well as to non-members. The ties are defined by regular exchange on agricultural climate change mitigation. Also, the strength of the ties and the type of relationship were assessed (datasets 3,4,8,9). Moreover, farmers were asked about some personal characteristics including attitudes towards climate change mitigation, assessment of the AgroCO2ncept project and their own mitigation behavior (datasets 1, 6). Farmers were furthermore asked to identify the perceived social influence of previously named contacts as well as (optionally) of additional persons (datasets 5, 10). For reasons of confidentiality, any comments, qualitative data or other personal information such as contact details of farmers were removed from the data.

The obtained interview data were matched with previously collected survey data^1^ on farmers’ adoption of mitigation measures, farmers’ behavioral characteristics as well as cantonal census data on farm structures and demographics (datasets 2, 7). In the survey, mitigation behavior was assessed by asking farmers to indicate which out of 13 selected mitigation measures they had adopted on their farm.

## 2 Experimental Design, Materials and Methods

**Fig. 1:**
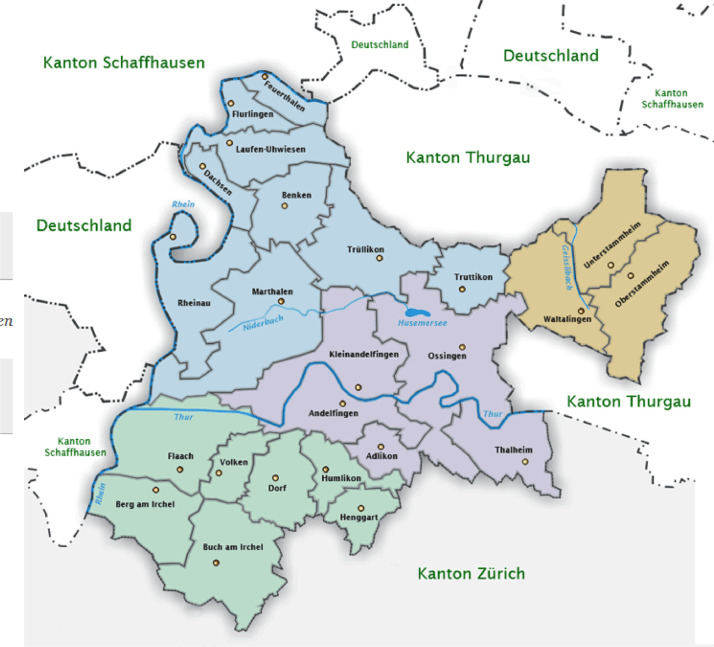
Map of the region of Zürcher Weinland including 24 municipalities (https://www.feuerthalen.ch/tourismus/umgebung/zuercher-weinland.html/323; see also [2]).

Out of the 50 interviewed farmers, 46 had previously participated in an online survey on behavioural factors of agricultural climate change mitigation, which was conducted by the authors in March and April 2019 [2]. The farms were located in the region of Zürcher Weinland, Canton Zurich in Switzerland. The region as shown in Fig. 1 includes 24 municipalities and is part of the political district of Andelfingen. Interview appointments were individually scheduled on the phone. AgroCO2ncept farmers were chosen based on their participation in the initiative. Additionally, we aimed to interview 25 farmers who did not participate and had ideally answered the online survey [2]. We asked approximately 60 farmers in the region out of which 25 were willing to participate.

**Fig. 2:**
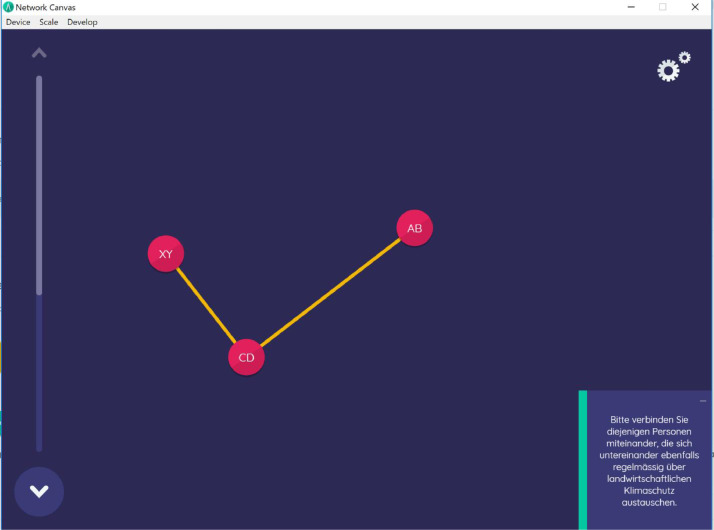
Alter-alter relations (“Please draw lines between the persons of whom you think they regularly exchange about agricultural climate change mitigation”).

**Fig. 3:**
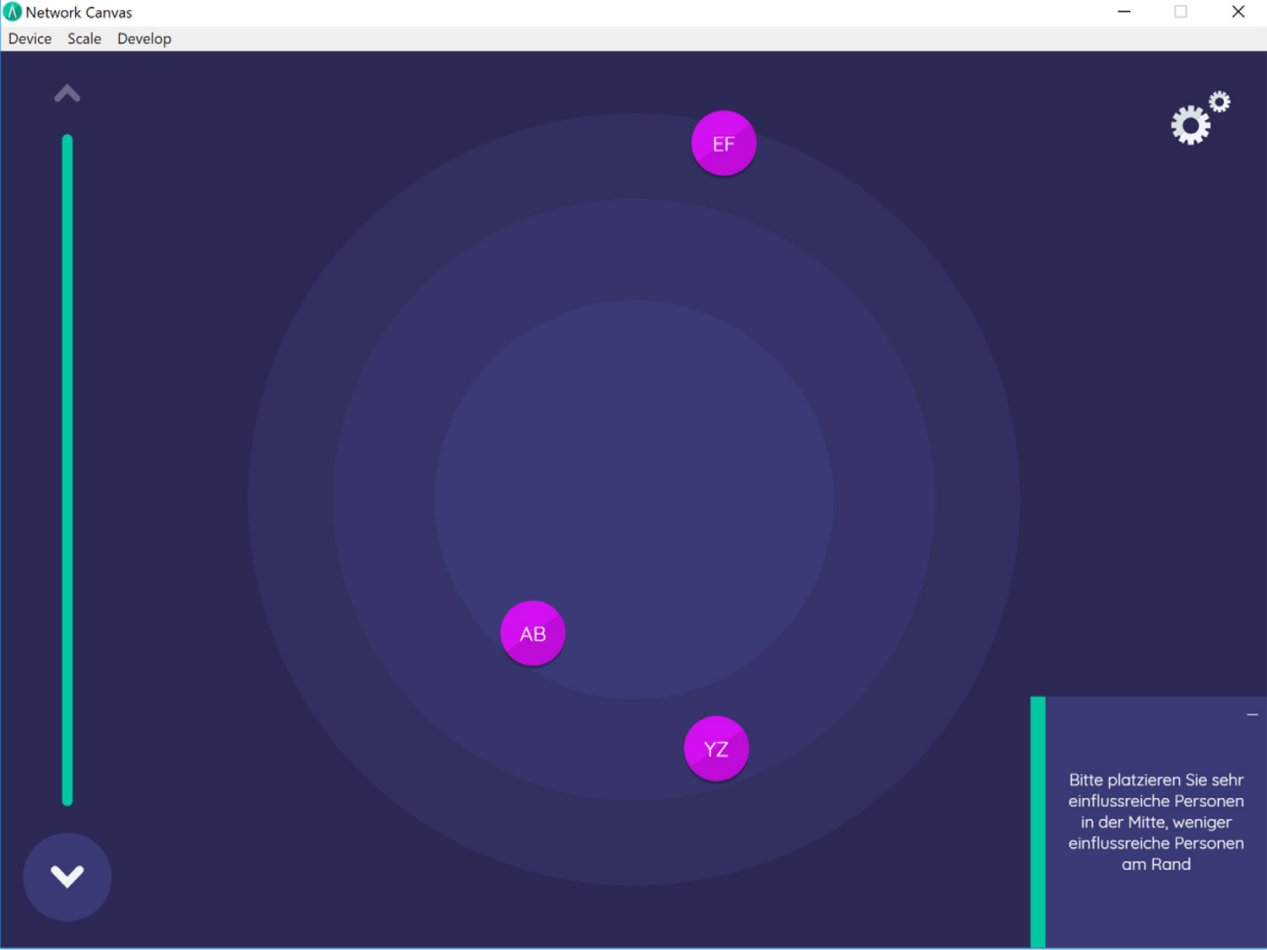
Influence ranking (“Please place people whom you perceive as very influential in the center of the concentric circle, less influential persons on the outer boundaries”).

The participating farmers were interviewed in November and December 2019 face-to-face by four trained interviewers on-site (usually on the farm). We used the free and open source network data collection software Network Canvas to design the questionnaire on tablets [3]. A touchscreen based data collection has been found to be more efficient than paper based methods of network data collection. The chosen Network Canvas software is a particularly modern, scientific and at the same time intuitive and user friendly tool [4]. Fig. 2 and Fig. 3 show examples of the touchscreen based questions.

Questions were read aloud by interviewers and simultaneously shown to farmers on the tablet. Most answers were inserted in the questionnaire by the interviewer. Some network related tasks (e.g. to draw ties between contacts or place persons on a concentric circle according to their influence) were directly executed by the interviewees on the tablet. Both questionnaires (for AgroCO2ncept participants and non-participants) were pre-tested for understanding, wording and user-friendliness with six students of agricultural sciences and three experts of social network research.

The questionnaire contained 29 questions for AgroCO2ncept participants and 25 questions for non-participants. On average, interviews lasted for about 30–40 min. The questionnaire for AgroCO2ncept farmers was structured in the following subsections:

i) Personal characteristics and AgroCO2ncept participation
ii) Agricultural climate change mitigation on the farm
iii) Name generator for regular exchange on agricultural climate change mitigation
iv) Name interpreter questions
v) Alter-alter relations
vi) Influential people

Similarly, the questionnaire for farmers not participating in AgroCO2ncept contained the following subsections:

vii) Personal characteristics and agricultural climate change mitigation on the farm
viii) Name generator for regular exchange on agricultural climate change mitigation
ix) Name interpreter questions
x) Alter-alter relations
xi) AgroCO2ncept project
xii) Contact to AgroCO2ncept participants
xiii) Name interpreter questions to AgrCO2ncept contacts
xiv) Influential people

### 2.1 AgroCO2ncept participants

i) *Personal characteristics and AgroCO2ncept participation*

Farmers participating in AgroCO2ncept were asked about the year in which they joined the initiative and how happy they were with it so far. Moreover, we asked whether they felt that their personal interests and opinions were sufficiently taken into account in the decision-making process of AgroCO2ncept and how they assessed the success of the project regarding the overall greenhouse gas reduction target. All questions had to be answered on 3- or 5-point Likert Scales.

ii) *Agricultural climate change mitigation on the farm*

We asked farmers to answer questions related to agricultural climate change mitigation. First, farmers were asked whether they considered to adopt (additional) mitigation measures on their farm. Next, we wanted to know how they assessed their success regarding the personal greenhouse gas reduction target committed to within AgroCO2ncept. Moreover, we asked farmers how important climate change mitigation was for their farming decisions in general and how that importance changed compared to 10 years ago. All questions had to be answered on 3- or 5-point Likert Scales.

iii) *Name generator for regular exchange on agricultural climate change mitigation*

Farmers were asked to indicate with whom they regularly exchanged about agricultural climate change mitigation. We here based on the existing literature on the important role of social networks in farmers’ adoption decisions, as for example shown in [5] and [6]. Interviewees (egos) were presented a roster with the names of all other AgroCO2ncept participants (alters). In order to choose a person as a contact, interviewees had to draw the name from the roster to an empty box on the touchscreen of the tablet. In addition, farmers had the option to name any other external person with whom they exchanged on the topic.

iv) *Name interpreter questions*

The following questions served to obtain information on the chosen alters and on the type and strength of the relationships [7]. Farmers were asked about the frequency of the exchange with the chosen contacts today and before joining AgroCO2ncept. Next, we asked them to indicate how they were currently related to them (e.g. friend, workmate, neighbor, family member etc.) and how they were related before joining AgroCO2ncept. We further asked participants how strongly every alter had influenced ego's decision to join the initiative. The following questions covered the alters’ perceived knowledge about agricultural climate change mitigation, how often the ego would ask the alters for advice on farming decisions and how much they would trust them. All questions had to be answered on 3- or 5-point Likert Scales.

v) *Alter-alter relations*

Here, farmers were asked to indicate whether the chosen alters would regularly exchange on agricultural climate change mitigation amongst each other. Knowing alter-alter relations allows for a deeper analysis of so-called “egocentric” networks (individual actors networks) [8]. To this end, interviewees had to randomly place the alters on the tablet and draw lines between those who were connected (see Fig. 2).

vi) *Influential people*

Lastly, we asked farmers to indicate whom they perceived as influential for decision-making of farmers in the region. Interviewees were presented the roster with all AgroCO2ncept participants again and could additionally name any external person who came to their mind in this context. In a next step, farmers were asked to rank the influence of the named persons on a concentric circle (see Fig. 3).

### 2.2 Farmers not participating in agroco2ncept

vii) *Personal characteristics and agricultural climate change mitigation on the farm*

First, farmers not participating in AgroCO2ncept were asked about the main production focus of their farm (in case no survey data was available for these farmers). Next, we asked whether they considered to adopt (additional) mitigation measures on their farm. As for AgroCO2ncept farmers in section *ii)* above, we asked about the importance of climate change mitigation for farming decisions today and 10 years ago.

viii) *Name generator for regular exchange on agricultural climate change mitigation*

Farmers were asked to indicate with whom they regularly exchanged about agricultural climate change mitigation. Interviewees (egos) could name any person (alter) by adding their name to a field on the tablet.

ix) *Name interpreter questions*

We asked for the same information about alters as presented in the AgroCO2ncept questionnaire above, see section *iv)*. The questions about influence on the decision to join AgroCO2ncept as well as about the relationship before joining were left out.

x) *Alter-alter relations*

See *v)* for AgroCO2ncept participants.

xi) *AgroCO2ncept project*

Farmers not participating in AgroCO2ncept were asked whether they knew the project and whether they had ever considered to join the project.

xii) *Contact to AgroCO2ncept participants*

Interviewees were presented a roster containing all AgroCO2nept participants, and asked to indicate whom they had regular contact with. In order to choose a person, they had to be clicked on the tablet.

xiii) *Name interpreter questions to AgrCO2ncept contacts*

Interviewees were asked to specify the type of relationship (e.g. friend, workmate, neighbor, family member etc.) and the frequency of exchange on agricultural climate change mitigation with the chosen AgroCO2ncept contact.

xiv) *Influential people*

See section *vi)* for AgroCO2ncept participants.

## Ethics Statement

All participating interviewees were thoroughly informed about the content and the scope of the study before participation. Thus, informed consent was obtained from the participants prior to the interviews. Participation was completely voluntary. Moreover, anonymity of the data is guaranteed by excluding all personal identifiable information of respondents.

## CRediT Author Statement

**Cordelia Kreft:** Conceptualization and design of questionnaires, conducting of interviews, data cleaning, writing manuscript; **Mario Angst:** Conceptualization, methodology, editing; **Robert Huber:** Conceptualization, pretests, editing, supervision; **Robert Finger:** Conceptualization, pretests, editing, supervision.

## Declaration of Competing Interest

The authors declare that they have no known competing financial interests or personal relationships which have or could be perceived to have influenced the work reported in this article.

## Footnotes

1 For a detailed description of the survey data used see [2].

## References

[1] AgroCO2ncept (2016). AgroCO2ncept Flaachtal - Eine regionale Initiative für den Klimaschutz in der Landwirtschaft. Ressourcenprojekt AgroCO2ncept Flaachtal - Antrag.

[2] Kreft C.S., Huber R., Wüpper D.J., Finger R. (2020). Data on farmers’ adoption of climate change mitigation measures, individual characteristics, risk attitudes and social influences in a region of Switzerland. *Data Brief*.

[3] Complex Data Collective (2016). Network Canvas: Software to Simplify Complex Network Data Collection. https://networkcanvas.com.

[4] Hogan B., Melville J.R., Phillips G.L., Janulis P., Contractor N., Mustanski B.S., Birkett M. (2016). Evaluating the paper-to-screen translation of participant-aided sociograms with high-risk participants. Proceedings of the 2016 CHI Conference on Human Factors in Computing Systems.

[5] Ramirez A. (2013). The influence of social networks on agricultural technology adoption. Procedia-*Soc. Behav. Sci.*.

[6] Matuschke I., Qaim M. (2009). The impact of social networks on hybrid seed adoption in India. Agric. Econ..

[7] Carolan B.V. (2013).

[8] Perry B.L., Pescosolido B.A., Borgatti S.P. (2018). Egocentric Network analysis: Foundations, methods, and Models.

